# Decoding paradoxical BOLD responses to transcranial ultrasound stimulation with concurrent optoacoustic magnetic resonance imaging

**DOI:** 10.1126/sciadv.adz1309

**Published:** 2025-10-31

**Authors:** Yi Chen, Zhenyue Chen, Hector Estrada, Irmak Gezginer, Hikari A. I. Yoshihara, Diana Kindler, Chunqi Qian, David C. Zhu, Shy Shoham, Daniel Razansky

**Affiliations:** ^1^ Institute for Biomedical Engineering and Institute of Pharmacology and Toxicology, University of Zürich, Zürich, Switzerland.; ^2^ Institute for Biomedical Engineering, Department of Information Technology and Electrical Engineering, ETH Zürich, Zürich, Switzerland.; ^3^ Institute of Precision Optical Engineering, School of Physics Science and Engineering, Tongji University, Shanghai, China.; ^4^ Department of Radiology, Michigan State University, East Lansing, MI 48824, USA.; ^5^ Department of Radiology and Gruss Magnetic Resonance Research Center, Albert Einstein College of Medicine, New York, NY 10461, USA.; ^6^ Neuroscience and Ophthalmology Departments, NYU Langone Health, New York, NY 10016, USA.; ^7^ Tech4Health Institute, NYU Langone Health, New York, NY 11101, USA.

## Abstract

Focused transcranial ultrasound stimulation (TUS) can affect neural activity with high spatial precision, advancing noninvasive neuromodulation toward targeted treatment of brain disorders. Direct monitoring of TUS responses is crucial for ensuring optimal outcomes. Blood-oxygenation-level–dependent (BOLD) functional magnetic resonance imaging has primarily been used for studying TUS effects in both human and nonhuman primate brains. However, the physiology and mechanisms underlying BOLD remain largely unknown due to its highly convoluted nature. To address these limitations, we developed a hybrid system for concurrent optoacoustic and magnetic resonance imaging of TUS (OMRITUS) to comprehensively characterize the hemodynamic changes in murine brains. Our findings reveal paradoxical negative BOLD signals in the activated cortical regions, coupled with increased total hemoglobin levels simultaneously monitored with optoacoustic tomography. Multispectral optoacoustic readings further demonstrated a stronger increase in deoxygenated versus oxygenated hemoglobin, suggesting a potential molecular basis for the negative BOLD responses. OMRITUS enables the study of complex TUS-hemodynamic interactions, paving the way for precise neuromodulatory interventions.

## Teaser

Concurrent optoacoustic and magnetic resonance imaging reveals paradoxical brain responses to ultrasound stimulation.

## INTRODUCTION

The ability to modulate brain activity noninvasively with high spatial and temporal precision provides transcranial ultrasound stimulation (TUS) with an enormous potential for the treatment of neurological disorders (*1*–*4*). The effects of TUS, ranging from behavioral changes to function improvement in both animals (*3*–*8*) and humans (*1*, *2*, *9*–*11*), have been observed using various methods. These include fluorescent calcium imaging to monitor neural activity (*8*, *12*), optical imaging to visualize hemodynamic responses (*13*), functional ultrasound imaging to assess blood flow changes (*14*, *15*), task or resting-state functional magnetic resonance imaging (rs-fMRI) to reveal increased functional brain network activity (*1*, *9*–*11*), and electrophysiology to measure electrical activity (*5*, *16*).

Despite these efforts, the effects of TUS on brain dynamics remain a subject of controversy both in human and animal studies. Focused ultrasound stimulation increases cerebral blood flow (CBF) and alters cortical hemodynamic responses, indicating potential neural excitation or increased vascular activity (*14*, *17*). Positive blood oxygenation level–dependent (BOLD) activity from focused ultrasound stimulation has been reported in three of the six human participants (*18*), while the application of focused ultrasound stimulation in the visual cortex elicited activations in both the sonicated brain area and its associated network regions (*19*). On the other hand, individual participant analysis revealed varied BOLD responses (positive, negative, or positive/negative) among participants (*20*). This variability in the effects of TUS may arise from differences in experimental parameters (*21*–*23*), such as stimulation intensity, frequency, and duration, as well as brain region targeted and the participants’ physiological state. Discrepancies between human and animal models, including differences in brain structure, hemodynamics, and experimental setups, add to the complexity. The resulting knowledge gap hampers optimization of the stimulation parameters needed for developing targeted interventions tailored for specific conditions (*24*). Therefore, an urgent need exists for advanced high-precision TUS tools with concurrent multiparametric imaging–based readings to enable systematic investigation of the processes by which TUS exerts its effects on the brain.

Now, functional magnetic resonance imaging (fMRI) is the most clinically accessible imaging modality for studying brain function and the one almost exclusively used for imaging TUS effects in both humans and nonhuman primate brains (*25*, *26*). Positive BOLD signals are generally associated with increased neural activity (*25*, *26*), whereas negative BOLD signals may indicate localized neural suppression and decreased metabolic demand (*21*) or, otherwise, a mismatched hemodynamic response to neural activity/metabolism (*27*, *28*). Besides the ambiguous nature of BOLD signals, fMRI falls short on delivering quantitative functional and molecular information with sufficient spatiotemporal resolution (*29*). Among potential complementary neuroimaging approaches, optoacoustic tomography (OAT) stands out with its ability to visualize hemodynamic changes and provide molecularly specific information with excellent spatial and temporal resolution (*30*). The technique uses the optoacoustic effect to produce high-contrast images based on differential optical absorption spectra of molecules and has an intrinsic capacity for spectroscopic differentiation between the variations of oxyhemoglobin (HbO), deoxyhemoglobin (HbR), and total hemoglobin (HbT) in a label-free manner. OAT has recently provided deeper insights into mechanisms underlying the BOLD signal (*30*–*33*). Hence, combining fMRI with OAT harnesses complementary strengths of both methods, creating a potent approach to disentangle TUS-induced hemodynamic changes across multiple physiological parameters and spatiotemporal scales.

Here, we introduce a fully hybridized system for concurrent optoacoustic and magnetic resonance imaging of TUS (OMRITUS) with holographic (multifocal) TUS capabilities to characterize hemodynamic changes comprehensively and unequivocally in the murine brain. As compared to a single-focus approach, the holographic TUS (hTUS) helps achieve broader regional coverage and greater flexibility in shaping the ultrasound field to match anatomical structures and slice thickness of fMRI measurements. Additionally, because the skull distorts ultrasound waves, leading to focal shifts and aberrations, hTUS helps to compensate for these distortions, thus improving the accuracy and robustness of stimulation. We validated the technique by targeting the retrosplenial cortex (RSC), which is widely accepted as a homologous region of the posterior cingulate cortex (PCC) in humans and primates (*34*–*37*). OMRITUS thus offers cutting-edge powerful multimodal capabilities to study complex TUS-hemodynamic interactions.

## RESULTS

### The multimodal OMRITUS platform

**Fig. 1.**
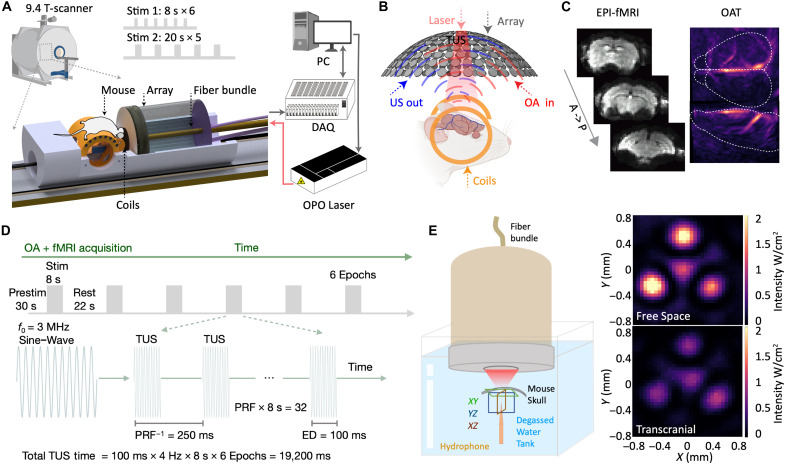
OMRITUS experimental design and data acquisition scheme. (**A**) Schematic of the hybrid system for concurrent magnetic resonance OAT imaging of TUS-induced hemodynamics in mouse brains. (**B**) Close-up of the stimulated and imaged area. US, ultrasound. (**C**) OMRITUS allows simultaneous volumetric fMRI and OAT acquisitions. A, anterior; P, posterior. (**D**) Illustration of the stimulation protocol applied to the right RSC of the mouse brain. The detailed TUS parameters are as follows: *f*_0_: center frequency of 3 MHz, emission duration of 100 ms, duty cycle of 40%, pulse repetition frequency (PRF) of 4 Hz, pressure of 0.52 MPa, stimulation duration of 8 or 20 s, interstimulation interval of 22 or 60 s, number of sessions of 6 for Stim1 and 5 for Stim2, 20 s for Stim1 baseline, and 30 s for Stim2 baseline. (**E**) Schematic of the setup for characterization hTUS using a hydrophone in a tank filled with degassed water (left). Ultrasound intensity maps measured in the free field (without the skull, top left) and transcranially (bottom right) at 3 MHz.

The hybrid OMRITUS platform is based on 9.4-T Bruker preclinical scanner (Fig. 1A). A magnetic resonance imaging (MRI)–compatible wide-angle 512-element spherical transducer array enables dual-modal fMRI-OAT imaging simultaneously with TUS emission (details in Materials and Methods) (Fig. 1, B and C). A 100-ms-long ultrasound pulse at a frequency of 3 MHz (Fig. 1D) is delivered into the mouse brain. Two stimulation paradigms were designed to modulate brain function (Fig. 1D; details in Materials and Methods). Because raw OAT sinograms can be corrupted during TUS emission (fig. S1A) or contaminated by Echo-Planar Imaging (EPI) eddy-current interference (fig. S1B), we developed a dedicated sinogram restoration algorithm to ensure the generation of artifact-free time-lapse volumetric OAT data (fig. S1 and movie S1). The volumetric OAT images were acquired at 50-Hz frame rate to ensure a sufficient number of noise-free frames for the analysis (details in Materials and Methods). After denoising, OAT-MRI coregistration was performed using a semiautomatic protocol, first in the subject space by aligning all EPI images to longitudinal relaxation time-weighted fast low-angle shot (FLASH) anatomical images (gray arrow in fig. S2). Then, the anatomical images from each subject were aligned to a mouse brain MRI template (light blue arrow in fig. S2) and OAT template (*38*), to which the OAT data from each subject was registered (dark blue arrow in fig. S2).

We characterized the hTUS delivery using a hydrophone to map the ultrasound field generated through an excised 6-week-old mouse skull (Fig. 1E). The holographic pattern generated at 3 MHz was preserved through the mouse skull with a reduced ultrasound intensity of ~40% (Fig. 1E and fig. S3) consistent with previous results (*39*). Guided by the MRI and OAT images, this multifocal ultrasound emission strategy successfully achieved focused TUS in the targeted region with high precision both in the unimpeded free field and transcranially (Fig. 1E and fig. S3).

### hTUS-evoked negative BOLD

**Fig. 2.**
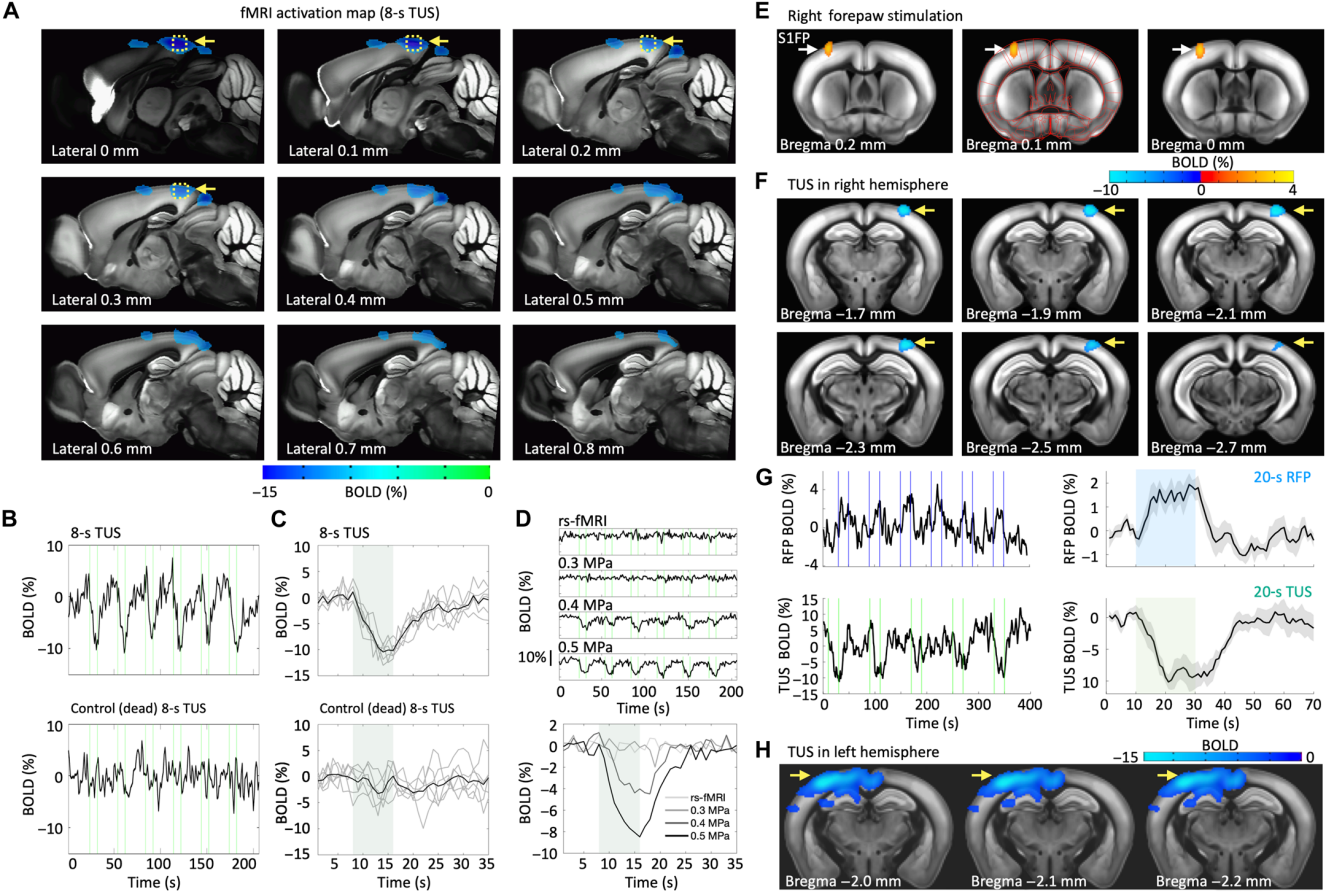
Characterization and validation of BOLD fMRI responses evoked by hTUS. (**A**) EPI-based fMRI activation map from mouse #1 (sagittal view) was overlaid on the Allen Mouse Brain Common Coordinate Framework (*48*). *P*_FDR_ < 0.05. (**B**) Time courses extracted from the regions activated with 8-s TUS [yellow dashed area in (A) for the in vivo and control (postmortem) trials from mouse #1]. (**C**) The corresponding averaged time courses for each epoch. (**D**) The time courses for each trial and each epoch of TUS pressure–dependent negative BOLD, including rs-fMRI. (**E**) Brain activation maps obtained from mouse #2 during electrical stimulation of the right forepaw. *P*_FDR_ < 0.01. (**F**) The corresponding brain activation maps obtained during TUS. *P*_FDR_ < 0.01. (**G**) The corresponding time courses extracted from regions activated by 20-s right forepaw stimulation (top) and by TUS (bottom) from mouse #2. (**H**) Brain activation maps obtained from mouse #3 when the TUS was targeting the left hemisphere. *P*_FDR_ = 0.001.

When applying 0.52-MPa focal pressure (measured transcranially by a hydrophone, as shown in Fig. 1E) combined with the holographic multipoint sonication strategy, we observed negative BOLD signals in the RSC (Fig. 2A). No notable BOLD changes were observed in the auditory or visual cortex, indicating that hTUS parameters used in this study did not evoke auditory or visual responses under a ketamine/xylazine anesthesia protocol (see the “Animal handling” section) (*40*). In principle, a decreased voxel intensity in the EPI-fMRI images may originate from increased/decreased neural activity (*25*, *27*), motion artefacts (*41*), or the effects of heat on temperature-sensitive magnetic resonance (MR) parameters such as the proton resonance frequency and transverse relaxation time (*42*, *43*). To better dissociate the various contributions, we performed control experiments with the same parameters after euthanasia of the animal. No apparent BOLD changes were detected with either a short hTUS duration of 8 s (Fig. 2, B to D) or a long duration of 20 s (fig. S4). Note that no OAT data were recorded in this particular experiment, which also helped eliminating its potential interference with the BOLD signal. Based on these results, dominant contributions of thermal effects, tissue displacement, and electronic noise to the negative BOLD signals were ruled out to ensure negligible artifactual fMRI readings in this study. During the experiment, animal body temperature was continuously monitored and maintained within the range of 36.5° to 37.5°C, with no associated changes observed during the TUS (fig. S5).

To further validate the negative BOLD signals that were evoked by TUS, we applied varying pressure levels of 0, 0.3, 0.4, and 0.52 MPa (table S1 and Materials and Methods). The amplitude of signal variations for 0.3-MPa sonication did not differ from resting-state fMRI recorded without hTUS, affirming that the negative BOLD signals were not caused by interference or noise from the setup (Fig. 2D). The negative BOLD signals began to appear only when TUS exceeded 0.4 MPa. Of note, the highest estimated pressure level was 0.52 MPa (fig. S3) considerably lower than the value needed to induce detectable thermal effects/mechanical movements, consistent with our previous (*39*) and others’ (*12*, *44*–*47*) findings. In addition, the anatomical images from three mice acquired with high-resolution rapid imaging with refocused echo (RARE) sequence indicated no visible tissue damage or abnormalities in the RSC 10 days after the hTUS experiments (fig. S6).

To test the specificity of the negative BOLD responses to TUS, we conducted further experiments involving electrical forepaw stimulation followed by TUS transmission in the same mouse. Positive BOLD responses were observed in the primary somatosensory cortex (S1) forepaw subregion, along with consistent negative responses in cortical areas targeted by TUS (Fig. 2, E to G). These results are consistent with our previously reported positive BOLD responses induced by electrical sensory stimulation when using the same hybrid MRI-OAT system but without ultrasonic waves emitted by the transducer (*31*). Notably, negative BOLD responses were also observed in a different mouse when targeting the left hemisphere (Fig. 2H), further substantiating reproducibility of the TUS-induced negative BOLD signals in the targeted regions.

### hTUS induces positive oxygenated and deoxygenated hemoglobin changes

**Fig. 3.**
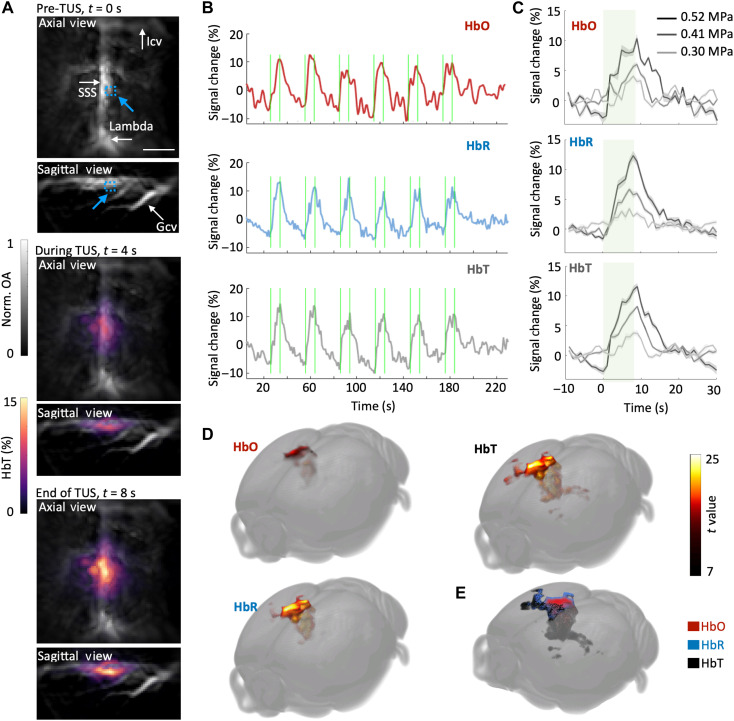
Multiparametric characterization of hTUS-evoked responses with spectroscopic whole-brain OAT. (**A**) Volumetric OAT image of the mouse brain spectrally unmixed to render maps of total (HbT) hemoglobin components before TUS (*t* = 0 s), during TUS (*t* = 4 s), and at the end of TUS (*t* = 8 s). Transverse (top) and sagittal maximum intensity projection views from a representative mouse are shown. Blue dashed box shows the region of interest (ROI). Scale bar, 2 mm. SSS, superior sagittal sinus; Icv, inferior cerebral vein; Gcv, vein of Galen. (**B**) Time courses of the HbO, HbR, and HbT components in response to hTUS. The ROI (0.4 mm by 0.4 mm by 0.4 mm) is indicated by the blue arrow in (A). (**C**) hTUS pressure–dependent changes in HbO, HbR, and HbT signals. The dark line with light shadow represents signal percentage change in response to 0.52-, 0.41-, and 0.3-MPa focal pressure as means ± SEM. (**D**) Activation maps resolved by the general linear model of the SPM12 software overlaid on the Allen Mouse Brain Common Coordinate Framework (*48*). The regions with the highest signal changes were consistent with the hTUS-focused regions. (**E**) Superposition of the HbO, HbR, and HbT activation maps on the mouse brain atlas.

Next, we performed spectroscopic whole-brain OAT to disentangle the hTUS-induced hemodynamic responses in the RSC. The easier accessibility and relatively large size of the RSC makes it a suitable target for precise focused TUS delivery, allowing for effective modulation with minimal risk to surrounding tissues. We applied the 8-s duration hTUS protocol depicted in Fig. 1D and observed pressure-dependent increases in the spectrally unmixed HbO, HbR, and HbT components. The volumetric OAT images before (*t* = 0 s), during (*t* = 4 s), and at the end of TUS transmission (*t* = 8 s) show a steady OAT signal increase in the targeted region (Fig. 3A), which is further confirmed by the signal time courses from a selected region (Fig. 3, B and C). The area with the highest signal changes was consistent with the RSC region targeted by hTUS. The averaged pressure-dependent responses for each epoch of hTUS (Fig. 3C) indicate that the hemodynamic responses become stronger as the applied focal pressure increases from 0.3 to 0.52 MPa. Superposition of the activation maps of HbO, HbR, and HbT on the Allen mouse brain atlas (Fig. 3, D and E) (*48*) further reveals a high spatial correlation between the hTUS-activated areas. We did not observe negative BOLD responses at 0.3 MPa, which can be attributed to the lower sensitivity of fMRI compared to that of OAT. Note that, to minimize the interference from fMRI to OAT measurement, EPI sequences were performed sequentially after OAT for this animal. Although high-pressure TUS may induce artifacts due to thermal effects (*39*) or tissue displacement (*15*), it is believed that such effects are transient and cannot account for the observed slow hemodynamic changes, particularly resulting from a relatively low pressure of 0.52 MPa.

### Multiparametric characterization of hTUS-evoked responses

**Fig. 4.**
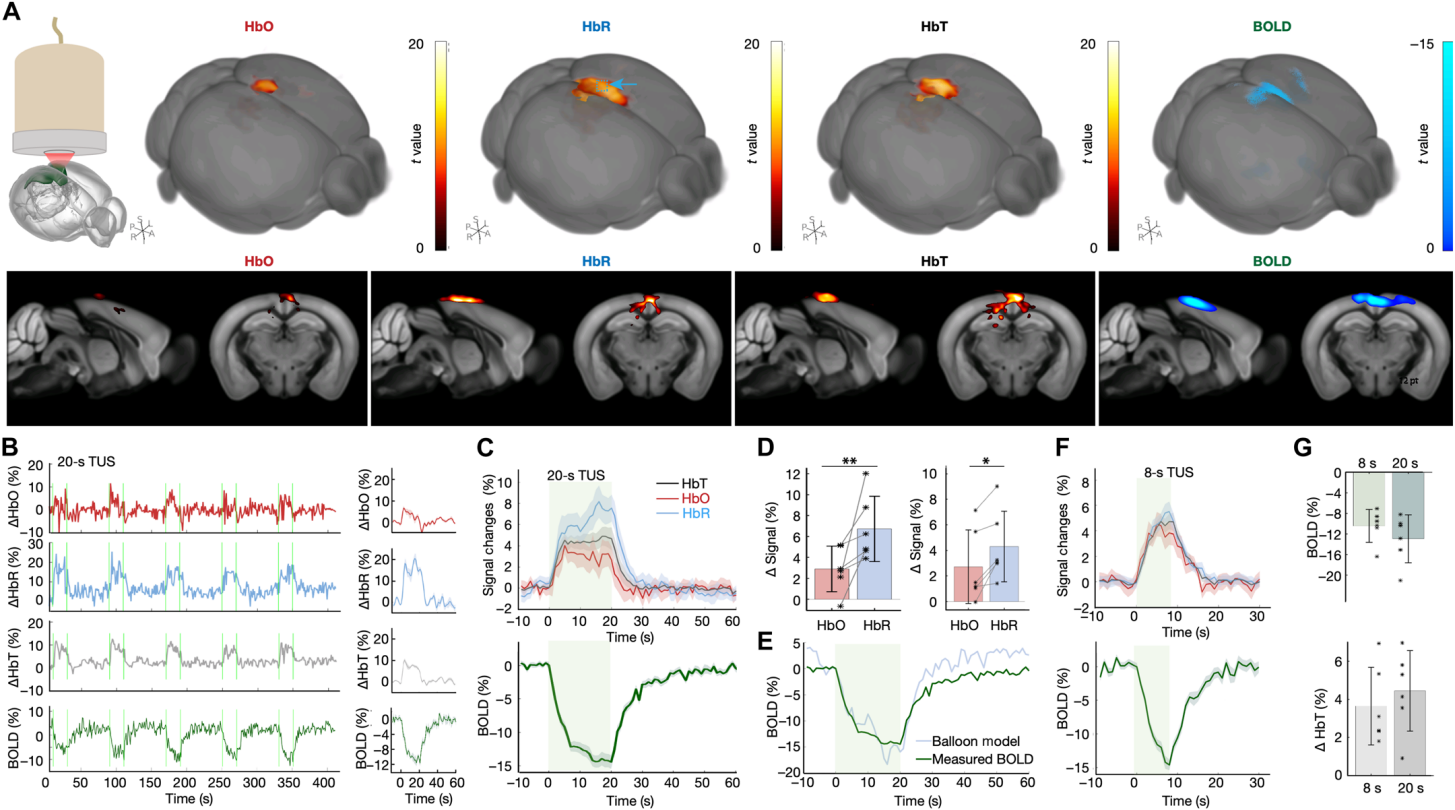
Group-level analysis of hTUS responses. (**A**) Location of the hTUS targeting (left). Multislice activation maps of the BOLD, HbO, HbR, and HbT hemodynamic components overlaid on the Allen Mouse Brain Common Coordinate Framework (*48*). Blued dashed box shows the ROI. A, anterior; P, posterior; L, left; R, right; S, superior; I, inferior. (**B**) Representative time courses of the four components from a single trial (left) and after averaging to each stimulation epoch (right, means ± SD). (**C**) Signal time courses for each epoch in response to 20-s hTUS averaged over six animals. (**D**) Mean percentage change of HbO and HbR in response to 20-s (left) and 8-s (right) hTUS. Paired *t* test, *P = 0.0128 and **P = 0.0411. (**E**) Balloon model–based prediction of BOLD induced by 20-s TUS. (**F**) Signal time courses for each epoch in response to 8-s hTUS averaged over six animals. (**G**) Averaged percentage change of HbT (left) and BOLD (right) from activated region (*n* = 6 animals).

To obtain more detailed temporal and spatial patterns, we simultaneously recorded hTUS-induced negative BOLD and positive multicomponent OAT responses, followed by the group and comparative analyses from *n* = 6 mice sonicated with the 8- and 20-s stimulation paradigms shown in Fig. 1A. The group activation maps show significant signal variations for the 20-s stimulation paradigm (Fig. 4A). We extracted the time courses for each hemodynamic component from a 0.4 mm–by–0.4 mm by–0.4 mm region of interest (ROI) within the activated region (Fig. 4B, left, and fig. S4) and calculated the averaged signal changes for each epoch (Fig. 4B, right). The time courses were subsequently averaged for all the stimulation cycles to compute the averaged time trace and fractional signal changes for HbO, HbR, HbT, and BOLD. The BOLD exhibited a clear negative trend after stimulation while HbO, HbR, and HbT increased (Fig. 4C). In addition, HbO, HbR, and HbT manifested different patterns: HbO responses were slightly lower with a longer hTUS duration; HbR displayed an opposite trend, while the magnitude of the HbT response was the same for the 8- and 20-s sonication (Fig. 4, C and F, top, and fig. S7). We then compared the temporal pattern of these three components (Fig. 4D). For 20-s sonications, the HbR component exhibited a stronger increase (6.7 ± 3.1%, means ± SD than the HbO (2.9 ± 2.2%), with the same behavior observed for 8-s sonication, i.e., 4.3 ± 2.8% increase for HbR versus only 2.7 ± 2.9% increase for HbO (paired *t* test, *P* = 0.0128 and 0.0411 for 8 and 20 s, respectively). The observed BOLD responses manifest a reasonable agreement with the predictions based on the extended balloon model of hemodynamic response (Fig. 4E) (*49*–*51*) when assuming a stronger coupling of the BOLD signal to the HbR component (λ_HbR_ = 1.7·λ_HbT_ in Eq. 1 in Materials and Methods), which is generally expected given its paramagnetic properties. Slightly stronger HbT (4.5 ± 2.1%) and BOLD (−12.9 ± 4.7%) responses were observed in 20-s hTUS trials as compared to that in 8-s hTUS (3.6 ± 2.0% for HbT and −10.5 ± 3.2% for BOLD) (Fig. 4F). Likewise, the HbR increased from 4.3 ± 2.8% to 6.7 ± 3.1% when the duration of hTUS increased from 8 to 20 s, implying an increased neuronal activity or more oxygen consumption by the cells (Fig. 4G). The HbO decay after 5 s of the hTUS application (Fig. 4, C and F, top, and fig. S7) indicates net deoxygenation of the region for the remaining duration of hTUS, which, together with the synergized effect of stronger HbR increase, may explain the negative BOLD.

## DISCUSSION

This work introduces a hybrid system for OMRITUS. It combines precise holographic ultrasound delivery with concurrent multimodal imaging of its induced brain activity. The multiparametric readings revealed negative BOLD responses accompanied by positive HbT changes in the stimulated region and surrounding brain areas (Fig. 4). Higher increase in the HbR signal compared to that in HbO was observed with spectroscopic OAT recordings, consistent with decreased blood oxygenation commonly associated with negative BOLD. Our concurrent multimodal findings thus offer a plausible interpretation of TUS-induced negative BOLD signals from the perspective of hemoglobin dynamics.

In rodent models, TUS has mainly been delivered to the motor cortex (*6*, *52*), hippocampus (*53*), somatosensory cortex of the hindlimb (*54*), thalamic nuclei (*46*, *55*), and visual cortex (*56*). However, no prior studies have investigated hemodynamic changes induced by TUS in rodent RSC. As a homologous region of PCC in humans and primates, RSC is a central hub in the default mode network (DMN) in rodents, which is prominently involved in baseline brain activity and is affected by numerous neurological and psychiatric conditions including consciousness, memory, and depression (*34*, *37*, *57*–*59*). Recent work has shown that low-intensity TUS on the PCC in humans increased corticospinal excitability (*9*) and the functional connectivity of the DMN (*10*). Our precise targeting restricted to the RSC (*60*–*62*) ensured negligible contaminating effects from collaterally activated brain regions. The rich vascularization in RSC and its relatively superficial location facilitates the monitoring of hemodynamic responses with OAT (*31*), providing clear and measurable hemodynamic changes. Future work should address the long-lasting effects mediated by TUS on RSC, such as whole-brain connectivity using rs-fMRI.

The BOLD contrast represents a change in the signal strength of brain water protons, mainly ascribed to the paramagnetic properties of venous blood (*63*). The balloon model first introduced by Buxton *et al.* (*49*, *50*) provided a way to simulate physiological BOLD responses based on assumption of a limited oxygen delivery at baseline and a slow recovery of venous blood volume after the stimulus, the so-called balloon effect. Follow-up studies revealed the nonlinearity of hemodynamic responses imaged with multimodal fMRI (*64*) and functional near-infrared spectroscopy (*51*), which provided more realistic and physiologically meaningful model estimation parameters. Here, we adopted the balloon model to predict the negative BOLD responses and rendered a good agreement with the experimental results showing stronger HbR signal changes upon TUS stimulation. Nonetheless, the accuracy of the model’s predictions should be interpreted with caution. While the balloon model is traditionally used to model positive BOLD responses to sensory stimulation, our case is distinct: The BOLD signal here likely reflects contributions from both brain activity and other vascular modulation effects induced by TUS (*65*). Additionally, the spatial and temporal resolution of our EPI acquisitions (1 mm and 1 s, respectively) imposes limitations on the modeling precision. Future effort will focus on integrating single-vessel fMRI and OAT to investigate molecular mechanisms underlying negative BOLD responses with greater specificity.

Several factors may potentially introduce unintended biological responses, and motion artifacts thus skew the results. For example, focused US has been reported to activate peripheral auditory pathways, leading to off-target activation across the brain (*66*, *67*). Localized thermal effects and mechanical strain on tissues can also mimic or obscure true neural activity and hemodynamic responses (*39*, *44*), potentially leading to false negative BOLD. To avoid confounds resulting from thermal accumulation and mechanical overstimulation, we used precisely targeted hTUS with low intensity, duty cycle, and pulse duration. Control trials in dead mice showed no negative BOLD changes, ruling out such confounding factors. Whole-brain fMRI used in this study aided in monitoring the auditory cortex and its associated projections: No responses were detected, confirming that our parameters did not elicit auditory activation. High-resolution anatomical images further effectively excluded major damage caused by TUS (fig. S5). No evidence of tissue damage was found by histological and immunohistochemical investigations (*n* = 12 animals) in our previous report (*39*) after using a peak pressure six times higher than what was used in the current study. In particular, considering the ultrasound intensity integrated over the sonication time (*68*) (100-ms pulses in this study versus 150 ms in the previous report), the effectively delivered sonication dose in this study was only a small fraction (2%) as compared to the ultrasound exposure in the previous study (*68*). Together, these results confirm the efficacy and safety of the TUS parameters used in this study.

The stronger HbR responses imply that the brain activation introduced by hTUS directly leads to oxygen consumption in the stimulated region. As the stimulation progresses, the increased blood flow results in a further increase of HbO, HbR, and HbT. The observed negative BOLD signals could be explained by its stronger dependence on the HbR and its higher increase in response to the stimulations, as revealed by the multispectral OAT measurements, although other vascular effects may play a key role in the activation cascade by altering metabolic support and oxygenation of neural tissues. Studies suggest that TUS can influence neuronal excitability through mechanical force, thermal effects, and cavitation (*8*, *12*, *69*). Other studies have indicated that TUS may selectively modulate the activity of excitatory and inhibitory neurons with specific stimulation frequencies (*21*, *46*, *70*). It has recently been reported that optimized ultrasound parameters could differentially induce excitation and inhibition even of deep-brain arousal centers such as midline thalamic nuclei (*45*). Beyond direct neuronal effects, TUS has also been implicated in vascular modulation. Studies have demonstrated that TUS can increase CBF (*17*) and persistently dilate cortical microvasculature without the use of microbubbles (*71*).

Based on our preliminary data and current understanding, we propose three plausible mechanisms underlying our observations, which are likely to involve a combination of effects rather than a single pathway. First, ultrasound has been reported to exert direct mechanical effects on blood flow, including the ability to transiently halt the movement of red blood cells (*65*). Consistent with this observation, our previous report (*72*) revealed a venous void during focused ultrasound stimulation. Second, holographic FUS has recently been reported to enhance stimulation efficacy by cooperatively recruiting distributed brain circuits (*73*). The network effect thus could potentially induce a net negative BOLD with both excitatory and inhibitory neurons. Last, ultrasound-induced neuromodulation has been shown to activate transient receptor potential ankyrin 1 channel in astrocytes (*74*, *75*). Given that astrocytes play a critical role in neurovascular coupling, including the regulation of CBF through modulation of vessel dilation, this mechanosensitive activation presents a compelling mechanism by which ultrasound may influence both neural and vascular dynamics.

OMRITUS is designed as a versatile platform with broad potential applications in neuroscience and biomedical research. It enables noninvasive mapping of brain circuitry by functional connectivity through MRI-guided localized stimulation in both healthy and diseased animal models. By incorporating spectroscopic OAT, it could further provide more specific insights into connectivity changes (*32*, *33*). Furthermore, OMRITUS facilitates the evaluation of tissue alterations and associated safety parameters following ultrasound treatment by using advanced imaging modalities, including diffusion imaging and susceptibility-weighted imaging. Because gadolinium-enhanced MRI remains the gold standard for evaluating blood-brain-barrier (BBB) integrity, while TUS can trap and modulate movement of microparticles, OMRITUS will also enable assessment of BBB opening, thus facilitating the development of safe, targeted, noninvasive drug delivery strategies in the brain. Last, OMRITUS could potentially be also integrated with fluorescence imaging (*39*) to assess neuro-astrocyte-vascular coupling or to identify cell-specific TUS effects. While TUS has been shown to affect both neuronal and vascular activity, advances in neuroimaging methodology are likely to provide deeper insights into the biophysical interactions between ultrasound waves, neurons, astrocytes, and vasculature, ultimately improving the efficacy and precision of TUS in both clinical and research settings. The recent development of calcium indicators targeting mechanosensitive (*8*, *12*) and heat-sensitive (*76*) ion channels can be applied to provide deeper mechanistic insights on the effects of TUS in the brain. Overall, OMRITUS could facilitate the study of rapid dynamic changes and complex interactions within the brain, leading to deeper insights into neurological conditions and the effects of therapeutic interventions.

## MATERIALS AND METHODS

### Hybrid OMRITUS system

The hybrid OMRITUS system uses the same spherical array transducer for TUS delivery and OAT-based functional monitoring (Fig. 1, A and B). The array has a radius of 40-mm and covers an angular aperture of 130° (solid angle of 1.15π sr) with its individual array elements having 5-MHz central frequency and >80% detection bandwidth at −6 dB. The excitation short-pulsed (<10 ns) light from an optical parametric oscillator laser (SpitLight, Innolas Laser GmbH, Germany) is delivered through an MRI-compatible fiber bundle (CeramOptec GmbH, Bonn, Germany), which is inserted into the 10-mm central aperture in the spherical array. The measured per-pulse energy is ~8 mJ at the skin surface. The 512-channel ultrasound array was integrated with a three-dimensionally (3D) printed animal holder containing a customized saddle radiofrequency (RF) coil (*31*) and inserted into in a 9.4-T Bruker BioSpec MRI scanner (BioSpec 94/20, Bruker BioSpin, Ettlingen, Germany). Three hundred eighty-four elements in total were used to generate the focused TUS emission and were operated in a passive mode for OAT data acquisition. A multichannel data acquisition system (DAQ, Falkenstein Mikrosysteme GmbH, Germany) was customized for the interleaved TUS emission and parallel OAT signal detection with a maximum digital temporal resolution of 5.5 ns and a sampling rate of 40 million samples per second. For single-wavelength OAT acquisitions, the laser wavelength is set to 800 nm. For multiwavelength acquisitions, the wavelength is rapidly swept on a per-pulse basis between five distinct wavelengths, namely, 700, 730, 755, 800, and 850 nm. The TUS is delivered at the center frequency of 3 MHz controlled by the DAQ.

To facilitate acoustic coupling, the volume between the active surface of the transducer and the mouse head is filled with heavy water (deuterium oxide) enclosed inside a customized polyetheretherketone cap attached to the transducer. The cap has a central 36-mm-diameter aperture covered with a thin layer of polyethylene membrane, which is optically and acoustically transparent and in contact with the mouse head. The simultaneous OAT and fMRI data acquisition is triggered by the laser output trigger signal and synchronized with the stimulation paradigm using an external trigger device (Pulse Pal V2, Sanworks, USA).

The mouse is placed in a prone position on a 3D printed cylindrically shaped holder with the nose pointing downward and the brain placed at the center of the spherical array geometry. This allows for maximizing the effective field of view (FOV) for OAT while also ensuring that TUS targets specific regions in the mouse brain with high accuracy. The two loops of the saddle RF coil are positioned on either side of the mouse head holder with their polarization perpendicular to the main magnetic field (fig. S1).

### Characterization of the holographic ultrasound field

To characterize the hTUS emission, we tested the holographic multipoint strategies depicted in Fig. 1E and fig. S3. The array was immersed in degassed water in a customized rectangular tank and a calibrated 75-μm-diameter hydrophone (Precision Acoustics, UK) placed in its focus. To characterize transcranial acoustic losses, a fresh skull extracted from a 6-week-old male mouse was mounted above the hydrophone’s tip. The parameters of TUS emission were controlled using a customized graphical user interface (GUI) implemented in MATLAB (2020, MathWorks Inc., USA) on a PC. The GUI was further adapted to control the OAT acquisition. We tested the focus size and pressure generated when the power was initially set to 3 V and characterized the relationships between the power amplifier voltage and US pressure distribution to determine the final parameters used for in vivo experiments in this study. Due to the small size of the US focal spot, which is even smaller than the fMRI voxel size, we implemented a multifocal strategy to activate three spots simultaneously. This characterization was crucial to accurately map locations of focal spots and pressure distribution in three dimensions (measured in the water tank using a hydrophone), both in free field and transcranially using a fresh skull of similar age placed between the hydrophone and the transducer (Fig. 1E).

### Animal handling

Eight C57BL/6J mice (Charles River Laboratories, Germany, 6 to 10 weeks) and six athymic female nude mice (Foxn1^nu^, Charles River Laboratories, Germany, 6 to 10 weeks) were used in this study. Animals were housed in individually ventilated, temperature-controlled cages under a 12-hour reversed dark/light cycle. Pelleted food (3437PXL15, CARGILL) and water were provided ad libitum. All experiments were performed according to the protocols (ZH060/22) approved by the Cantonal Veterinary Office Zürich, as required by the Swiss Federal Act on Animal Protection.

For the in vivo experiments, the animals were initially anesthetized by intraperitoneal injection of a mixed bolus of ketamine (100 mg/kg of body weight, Pfizer, Switzerland) and xylazine (10 mg/kg of body weight, Bayer, Germany) in two steps with a 5-min gap to prevent cardiac depression. The anesthesia was maintained by using a lower concentration mixture of ketamine (25 mg/kg of body weight) and xylazine (1.25 mg/kg of body weight) injected intraperitoneally every 45 min. The fur on the mouse head was removed using shaving cream and the scalp and skull of both C57BL/6J mice and nude mice were kept intact. The mouse was then transferred to the OMRITUS system and positioned on the customized mouse bed in a prone position with the nose pointing downward using a customized stereotaxic frame (Fig. 1A). To ensure optimal acoustic coupling through the transparent polyethylene film and to minimize signal distortion in the MRI data, ultrasound gel was mixed with heavy water and applied on the head of the mouse. The volumetric OAT reconstruction rendered on-the-fly in the customized GUI was used to navigate and adjust the exact position of the TUS focus. During the setup and imaging session, a mixture of oxygen/air (0.2/0.8 liters min^−1^) was delivered through a breathing mask. Heart rate and SpO_2_ were monitored using an MRI-compatible mouse paw pulse oximeter connected to a PhysioSuite (Kent Scientific Corporation, USA). The animal’s body temperature was continuously monitored and maintained within a physiological range of 36.5° to 37.5°C throughout the experiment. This was achieved using a rectal temperature probe (MLT415/DL in combination with LabChart software and a PowerLab 16/30 (all from AD Instruments, New Zealand), along with thermal regulation via temperature-controlled water circulating (Lauda E100 Thermostat, Gemini Sustainable Laboratory Equipment, The Netherlands) through tubing surrounding the animal. The respiration rates were continuously measured with an MRI-compatible pneumatic pillow (SA Instruments, USA) and the same AD Instruments system (LabChart software and a PowerLab 16/30, AD Instruments, New Zealand) used for the temperature measurements.

### OAT data acquisition and analysis

For the functional (spectroscopic) OAT data acquisition, we used a multiwavelength illumination protocol at 700, 730, 755, 800, and 850 nm. For each wavelength in both protocols, the concurrent fMRI acquisition using EPI sequences induced corrupted frames due to eddy currents or mechanical noise from the gradient coils. The corrupted frames were first identified in the acquired sinograms by subtracting the nominal mean values of each channel, as previously described (fig. S1) (*31*). The spectroscopic (five wavelengths) volume rate was then down sampled to 1 Hz to restore the sinogram and match the fMRI volume rate. During this process, refined sinograms were created through pixel-wise processing, and outliers were discarded before averaging consecutive frames (movie S1). Next, the raw signals from each of the 384 channels were bandpass (0.1 to 8 MHz) filtered, and image reconstruction was carried out using a GPU-based filtered back-projection algorithm (*31*). To achieve accurate OAT reconstructions, we applied a dual-speed-of-sound algorithm (*30*, *31*) by considering the speed of sound values of ~1400 and 1530 m/s in the heavy water medium and mouse tissues, respectively. To obtain a better balance of processing time and quality of registration to the template, the voxel size was reset to 100 μm by 100 μm by 100 μm, and the FOV was adjusted to 8 mm by 8 mm by 4 mm. After this step, images were normalized using laser pulse energy readings at corresponding wavelengths and further normalized with an exponential light attenuation function to compensate for signal intensity decay with depth. Last, linear spectral unmixing (*30*) was performed to determine the distribution of HbO and HbR in the vascular structure of the mouse brain. HbT was calculated as the sum of HbO and HbR. The first 5 s of the OAT scans, corresponding to the laser warming-up state and the dummy scans in fMRI data, were discarded before preprocessing.

### MRI image acquisition

All MRI images were acquired using a 9.4-T/20-cm horizontal bore small animal MR systems using custom-made RF coils with ParaVision 6.0.1 software. Adjustments to echo spacing and symmetry and B0 compensation were set up first for EPI sequence. Magnetic field homogeneity was optimized by global shimming for anatomical images and followed by the fast automatic shimming technique by mapping along projections (FASTMAP) shimming protocol for EPI sequences. A 10-min rs-fMRI was acquired before and after the TUS session. The task-fMRI during the TUS session was acquired with a gradient-echo EPI sequence with the following parameters: echo time (TE) of 11.5 ms, repetition time (TR) of 995 ms, FOV of 1.92 cm by 1.92 cm by 1.92 cm, matrix size of 48 × 48 × 48, and spatial resolution of 0.4 mm by 0.4 mm by 0.4 mm. The detailed TUS parameters are as follows: frequency, 3 MHz; pulse duration, 100 ms; duty cycle, 40%; pulse repetition frequency, 4 Hz; pressure, 0.52 MPa; stimulation duration, 8 s; interstimulation interval, 22 or 60 s; number of sessions, 6 for Stim1 and 5 for Stim2; and 20 s for Stim1 baseline and 30 s for Stim2 baseline. For the fMRI acquisition with an 8-s duration of hTUS, the paradigm (block design) for each trial consisted of 30 TR prestimulation scans, 8 TR scans during stimulation, and 22 TR poststimulation scans with a total of six epochs. The total number of TRs is 210 TR (see details in Fig. 1A). For the fMRI acquisition of 20-s duration of hTUS, the paradigm (block design) for each trial consisted of 30 TR prestimulation scans, 20 TR scans during stimulation, and 60 TR poststimulation scans with a total five epochs. The total number of TRs is 430 (see Fig. 1A). For the fMRI acquisition of 20-s duration electrical stimulations on the right forepaw, the paradigm (block design) for each trial consisted of 40 TR prestimulation scans, 20 TR scans during stimulation, and 40 TR poststimulation scans with a total of six epochs. The total number of TRs was 415. The parameters for the paw stimulation are as follows: 0.5-ms duration and 0.5-mA intensity were applied to the right forepaw at 4-Hz stimulus frequency. A stimulus isolator device (Model A365R, World Precision Instruments, USA) fed by an external trigger (Pulse Pal V2, Sanworks, USA) was used to generate electrical stimulation. Subsequently, we applied a higher resolution 2D FLASH sequence to acquire 11 coronal slices with the same geometry as the fMRI images, as an anatomical reference, with the following parameters: flip angle of 30°, FOV of 20 mm by 10 mm, matrix size of 160 × 80, 11 slices from anterior to posterior, slice thickness of 0.7 mm, TR of 500 ms, and TE of 2.1366 ms, yielding an effective spatial resolution (0.125 mm by 0.125 mm by 0.7 mm) for the registration.

The anatomical MRI images for post-TUS with higher resolution in fig. S6 were acquired on a 7-T MRI scanner (PharmScan, Bruker BioSpin, Ettlingen, Germany) using a CryoProbe with ParaVision 6.0.1 software. We used a 2D RARE sequence to acquire 19 coronal slices with the following parameters: TR of 2500 ms, echo time of 34.3 ms, bandwidth of 32894.7 Hz, FOV of 15.4 mm by 15.4 mm, matrix size of 256 × 192, resolution of 60 μm by 80 μm, slice thickness of 0.4 mm, slice gap of 0 mm, RARE factor of 6, and average of 6.

### TUS-evoked fMRI data analysis

Functional MRI images were analyzed using Analysis of Functional NeuroImages (AFNI; NIH, USA) and Statistical Parametric Mapping software package (SPM12; Functional Imaging Laboratory, Welcome Trust Centre for Human Neuroimaging, University College London, UK). The semi-autoregistration (Fig. 1C) used MATLAB integrated with the open-source SPM12. For the stimulus-evoked fMRI, the preprocessing procedures followed that commonly used protocol (*30*, *31*), including despiking (3dDespike in AFNI), slice-timing correction (3dTshift in AFNI), motion-correction (3dvolreg in AFNI), spatial smoothing (3dmerge in AFNI, 0.5-mm full width at half maximum Gaussian kernel), image registration (afni_proc.py in AFNI) to anatomical images acquired in the same orientation with the same geometry, and time course normalization (3dcalc in AFNI) for each animal. Six motion parameters obtained in the motion correction step (3dvolreg in AFNI) were also regressed to further reduce motion artifacts. A high-pass filter with a cut-on frequency of 1/200 Hz was used to remove slow signal drifts. Then, the anatomical FLASH images were registered to the template across animals and generated the registration matrix, which was used to align EPI fMRI datasets to the same template. The averaged baseline signal of EPI images, i.e., pre-hTUS, was normalized to 100 for statistical analysis of multiple animals. The regression analysis of the hemodynamic response function (HRF) was based on the BLOCK function of the linear program 3dDeconvolve in AFNI. BLOCK (*d*, 1) computes a convolution of a square wave of duration d and makes a peak amplitude of block responses of 1. To compute the evoked BOLD changes, the time courses of the BOLD signals in Figs. 2 and 4 and fig. S4 were extracted from the TUS focal region having substantial activations. Note that the first 5 s of the fMRI were discarded before preprocessing to remove the dummy scans from fMRI before EPI sequences reached steady state.

The balloon model to predict fractional BOLD changes as a function of normalized blood volume (HbT) and normalized deoxyhemoglobin content (HbR) is formulated as follows (*50*, *51*)

$$\text{BOLD}=V_{0}\cdot \left[\lambda_{\text{HbR}}\;\left(1-\mathrm{HbR}\right)-\lambda_{\text{HbT}}\;\left(1-\text{HbT}\right)\right]$$ (1)

where *V*_0_ is baseline blood volume fraction, λ_HbR_ is the weight for HbR changes, and λ_HbT_ is the weight for blood volume (HbT) changes.

### Statistical analysis

All statistical analysis was done using MATLAB and AFNI. The activation maps from different channels (i.e., HbO, HbR, HbT, and BOLD) were obtained for each animal with general linear model analysis. The first-order canonical basis set with the convolution of HRF and the TUS paradigm was used as a regressor. The six motion parameters obtained from preprocessing of motion correction were used to regress out in both fMRI and OAT analysis to reduce motion artifacts. A high-pass filter with a cut-on frequency of 1/200 Hz was used to remove slow signal fluctuations. The activation maps for six animals were obtained by using 3dttest++ in AFNI (*P* < 0.001) and false discovery rate (FDR)–corrected cluster-size thresholds. The relative percentage change of each channel for each voxel was calculated by assuming the baseline to be the average signal in a 5-s prestimulation window. The activation time courses were obtained by averaging all the stimulation epochs (Fig. 4, C and F) from all animals. The averaged amplitude of HbT, HbO, HbR, and BOLD in Fig. 4 (C and F) was defined as the mean value of 5 to 8 s for 8-s duration of TUS and 5 to 20 s for 20-s duration of TUS. A paired *t* test was used for generating Fig. 4D.
