# Supplementary material for: Decoding paradoxical BOLD responses to transcranial ultrasound stimulation with concurrent optoacoustic magnetic resonance imaging
Source: Sci Adv. 2025 Oct 31;11(44):eadz1309. doi: 10.1126/sciadv.adz1309 (PMC12577710; doi:10.1126/sciadv.adz1309)
Supplement: Supplementary file 1 — Figs. S1 to S7 Table S1 Legend for movie S1 [file sciadv.adz1309_sm.pdf]

Science Advances
AAAS

Supplementary Materials for

Decoding paradoxical BOLD responses to transcranial ultrasound stimulation with concurrent optoacoustic magnetic resonance imaging

Yi Chen *et al.*

Corresponding author: Daniel Razansky, daniel.razansky@uzh.ch; Shy Shoham, shoham@nyu.edu

Sci. Adv. **11**, eadz1309 (2025)
DOI: 10.1126/sciadv.adz1309

The PDF file includes:

Figs. S1 to S7
Table S1
Legend for movie S1

Other Supplementary Material for this manuscript includes the following:

Movie S1

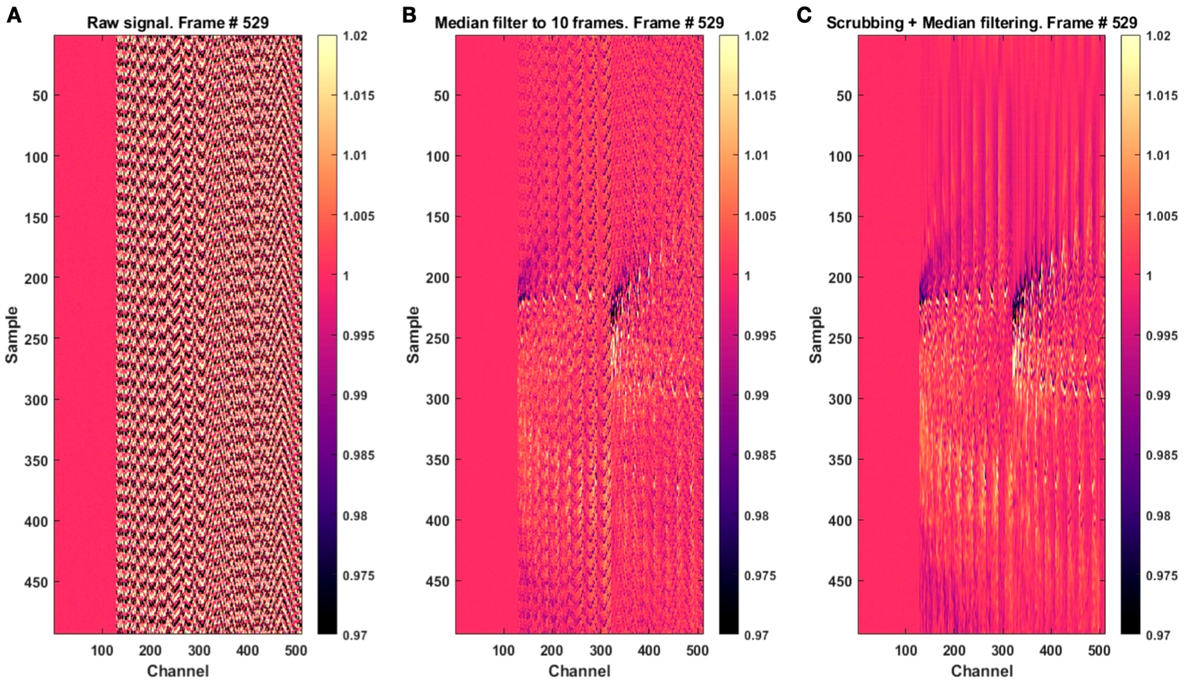

Fig. S1. Denoising of OAT data contaminated by TUS and EPI sequences. A. The raw signal shows interrupted frames during TUS. B. The signals are cleaned after median filtering. C. The denoised frame after snubbing and median filtering.

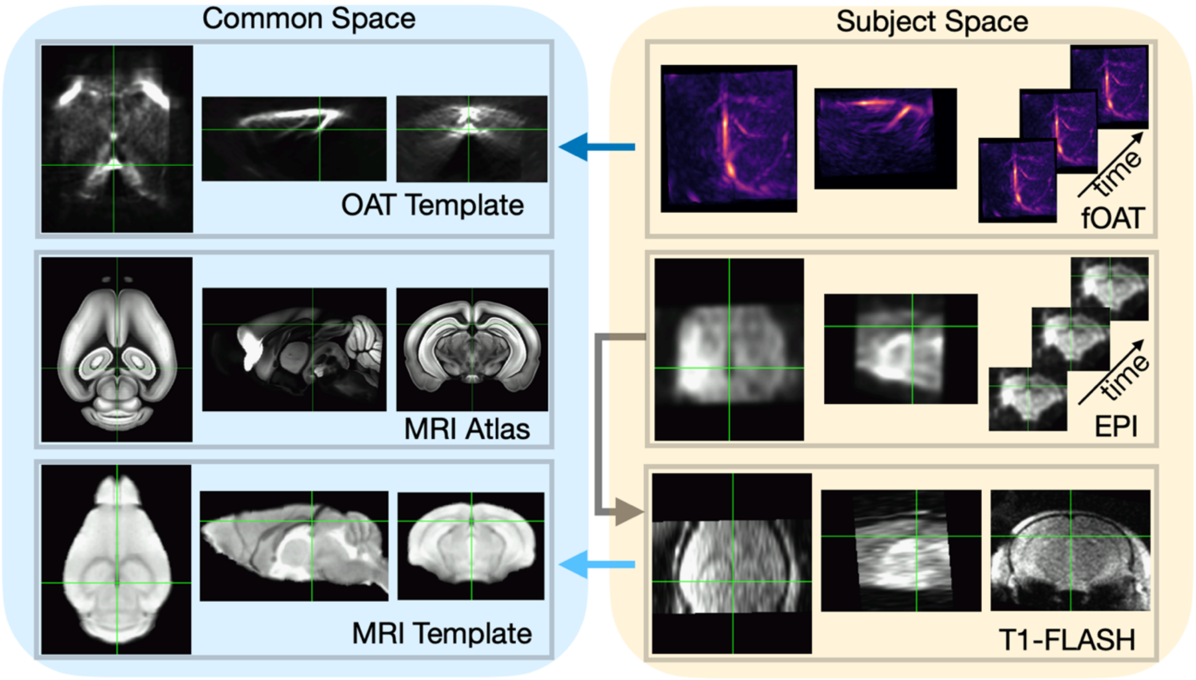

Fig. S2. Flowchart displaying the main data analysis pipelines used in this study. Arrows indicate registration directions. More details are provided in Methods.

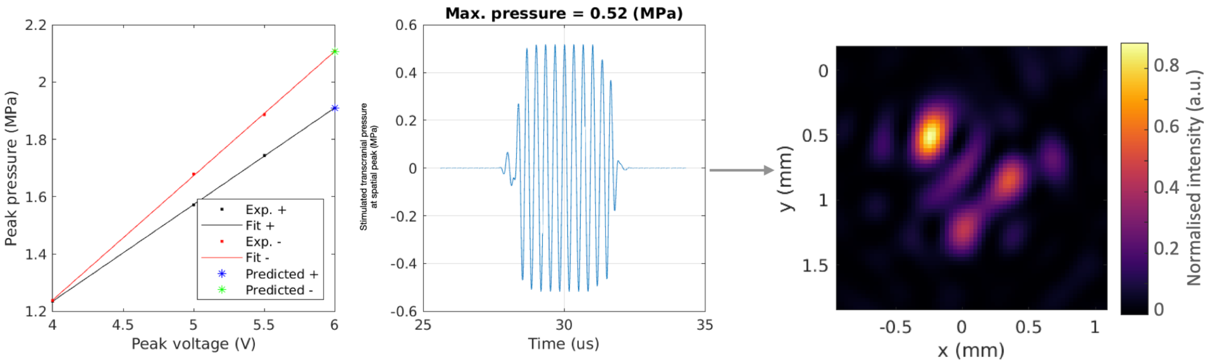

Fig. S3. Characterization of transcranial pressure based on stimulation parameters. Results show that FUS was delivered at maximal pressure of 0.3 MPa, 0.41 MPa, and 0.52 MPa in this study.

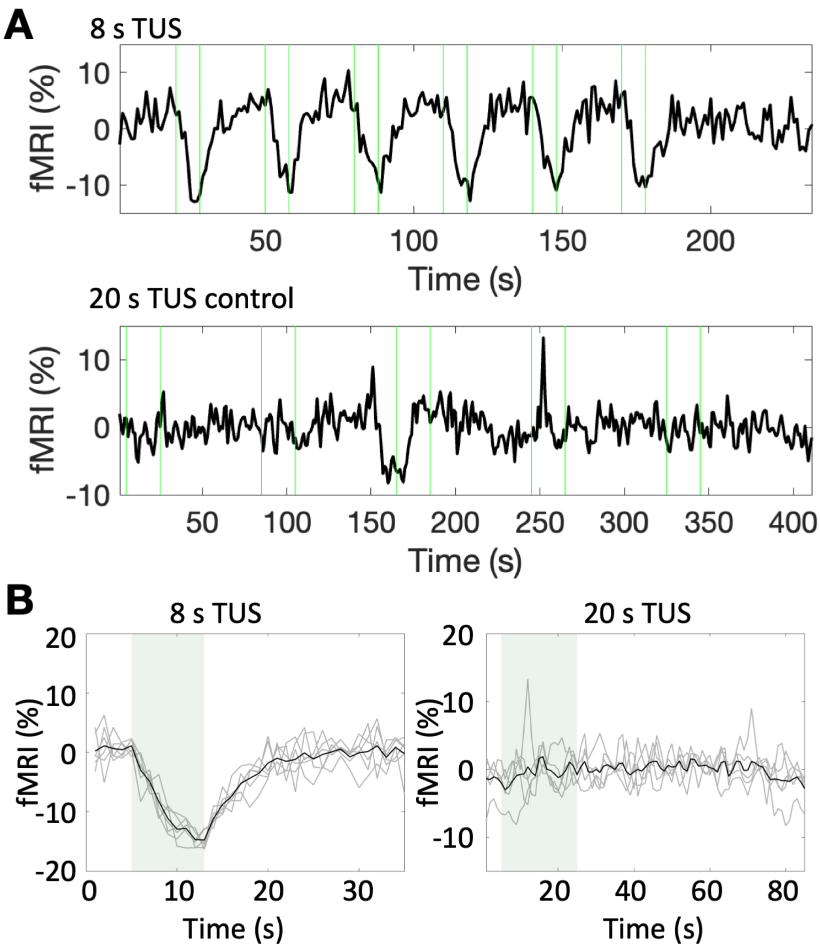

Fig. S4. Extracted time courses with 8 s and 20 s TUS stimulation from another representative mouse. A. EPI time courses extracted from activated regions of the same mouse with 20 s and 8 s TUS during normal and control (dead) trials, respectively. B. Averaged time courses for each epoch shown in A.

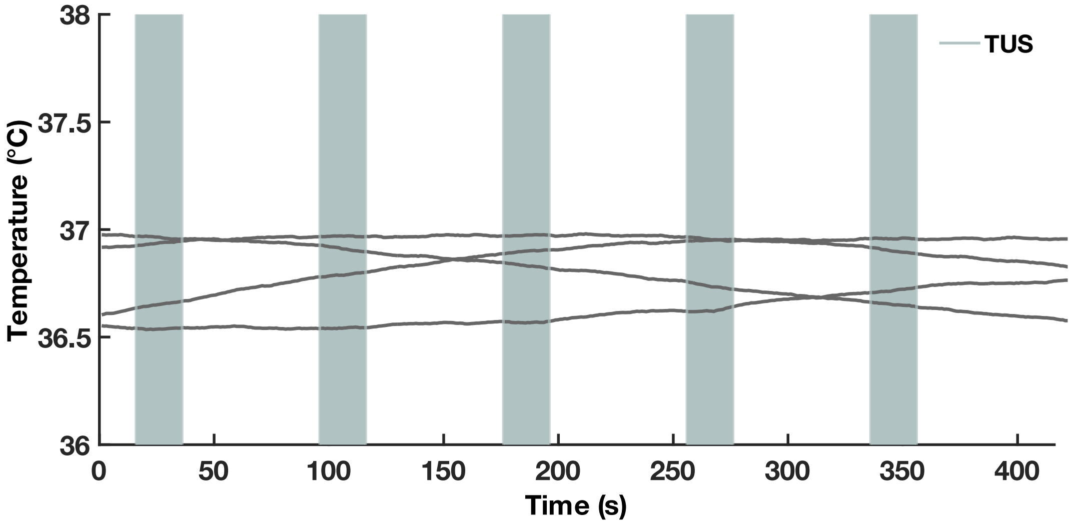

Fig. S5. Body temperature changes during TUS across four trials from three mice. Temperature was monitored and maintained within the range of 36.5–37.5°C.

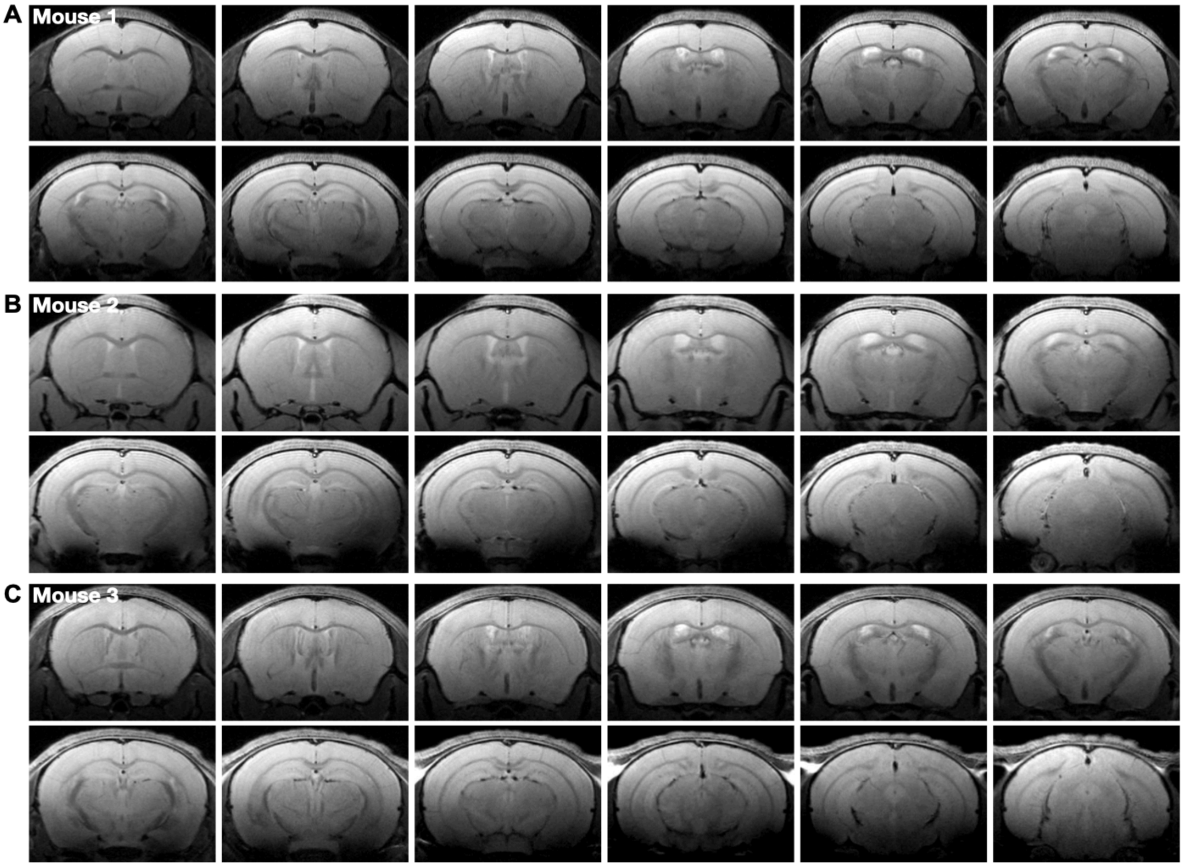

Fig. S6. High-resolution RARE anatomical images from three mice indicate no visible tissue damage or abnormalities in the RSC after TUS experiments. MRI images were acquired by a 7T MRI scanner (BioSpec, Bruker BioSpin, Germany) using a CryoProbe with the software ParaVision 6.0.1. We used the 2D RARE sequence to acquire 19 coronal slices with the following parameters: Repetition Time, 2500 ms; Echo Time, 34.3 ms; Bandwidth = 32894.7 Hz, FOV = 15.4 mm × 15.4 mm, Matrix Size = 256 × 192, in Plane Resolution = 60 µm × 80 µm, Slice Thickness = 0.4 mm, Slice Gap: 0 mm; RARE Factor = 6, Averages = 6.

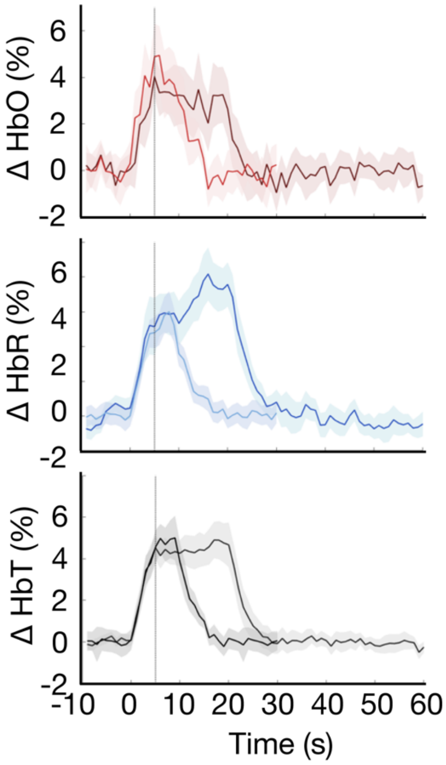

Fig. S7. Comparison of HbO, HbR, and HbT responses from 8 s TUS (lighter shade) and 20s TUS (darker shade) (Mean ± SEM). The gray lines in all three panels indicate 5 s after stimulation. Note that 8 s TUS showed a faster recovery to baseline.

	CF	PD	DC	PRF	Pressure	SD	ISI	Epochs	Baseline
Stim 1	3 MHz	100 ms	40%	4 Hz	0.52 MPa	8 s	22 s	6	30 s
Stim 2	3 MHz	100 ms	40%	4 Hz	0.52 MPa	20 s	60 s	5	30 s

Supplementary Table 1. TUS parameters for the two stimulation conditions. CF, center frequency; PD, pulse duration; DC, duty cycle; PRF, pulse repetition frequency; SD, stimulation duration; ISI, inter-stimulation interval.

Supplementary Movie 1. Denoising of OAT data contaminated by TUS and EPI sequences. Left: The raw signal shows interrupted frames during TUS. Middle: The signals are cleaned after median filtering. Right: The denoised frame after snubbing and median filtering.
